# Prognostic Value of Androgen Receptor Splice Variant 7 in the Treatment of Metastatic Castration-Resistant Prostate Cancer: A Systematic Review and Meta-Analysis

**DOI:** 10.3389/fonc.2020.562504

**Published:** 2020-11-30

**Authors:** Jiaxin Wang, Yucong Zhang, Chao Wei, Xintao Gao, Penghui Yuan, Jiahua Gan, Rui Li, Zhuo Liu, Tao Wang, Shaogang Wang, Jihong Liu, Xiaming Liu

**Affiliations:** ^1^ Department of Urology, Tongji Hospital, Tongji Medical College, Huazhong University of Science and Technology, Wuhan, China; ^2^ Department of Geriatrics, Tongji Hospital, Tongji Medical College, Huazhong University of Science and Technology, Wuhan, China

**Keywords:** androgen receptor splice variant 7, metastatic castration-resistant prostate cancer, prognosis, androgen receptor signaling inhibitors, chemotherapy

## Abstract

**Background:**

The prognostic value of androgen receptor splice variant 7 (AR-V7) for the treatment response of metastatic castration-resistant prostate cancer (mCRPC) remains unclear. In this study, we aimed to synthesize relevant studies that assessed the prognostic value of AR-V7 status for the treatment response of mCRPC patients treated with androgen receptor signalling inhibitors (ARSis) and chemotherapy.

**Methods:**

We searched the PubMed, Embase, and MEDLINE databases by using the keywords *AR-V7* and *prostate cancer* to identify relevant studies published before 25 September 2019. The main outcomes were prostate-specific antigen (PSA) response, progression-free survival (PFS), and overall survival (OS). Pooled odds ratios (ORs) and hazard ratios (HRs) with 95% confidence intervals (CIs) were calculated using a random effects model. The quality of the included studies was assessed using the Newcastle-Ottawa Quality Assessment Scale.

**Results:**

A total of 1,545 patients from 21 studies were included. For the mCRPC patients treated with ARSis, AR-V7-positive patients had a lower PSA response rate (OR 6.01, 95% CI 2.88–12.51; *P* < 0.001), shorter PFS (HR 2.56, 95% CI 1.80–3.64; *P* < 0.001) and shorter OS (HR 4.28, 95% CI 2.92–6.27; *P* < 0.001) than AR-V7-negative patients. Although AR-V7-positive patients treated with chemotherapy also had a lower PSA response rate (OR 2.23, 95% CI 1.38–3.62; *P* = 0.001) and shorter OS than AR-V7-negative patients (HR 1.60, 95% CI 1.02–2.53; *P* = 0.043), there was no significant difference in PFS (HR 1.05, 95% CI 0.74–1.49; *P* = 0.796) between these groups. Furthermore, AR-V7-positive patients receiving ARSis had a shorter median OS than those receiving chemotherapy (HR 3.50, 95% CI 1.98–6.20; *P* < 0.001); There was no significant difference among AR-V7-negative patients (HR 1.30, 95% CI 0.64–2.62; *P* = 0.47).

**Conclusions:**

AR-V7 is a potential biomarker of treatment resistance in mCRPC patients. AR-V7-positive mCRPC patients had poorer treatment outcomes than AR-V7-nagetive patients when treated with ARSis. AR-V7-positive patients have better outcomes when treated with taxane than ARSis. Furthermore, the ability of AR-V7 status to predict treatment outcomes varies from different detection methods. The detection of AR-V7 before treatment is important for the selection of treatment modalities for mCRPC patients.

## Introduction

Prostate cancer is one of the most common cancers among male patients in the world (1). Most patients will progress to castration-resistant prostate cancer (CRPC) during primary androgen deprivation therapy (2). The use of androgen receptor signalling inhibitors (ARSis) and chemotherapy by taxanes are the standard-of-care for metastatic CRPC (mCRPC) (3). Moreover, recently, ARSis and docetaxel have been approved for newly diagnosed metastatic hormone-sensitive prostate cancer (HSPC), showing promising prospects for the treatment of prostate cancer (4, 5). However, some patients showed primary resistance at the beginning of treatment with ARSis. Moreover, most mCRPC patients treated with ARSis ultimately suffer tumour progression, and the reasons for resistance to ARSis remain unclear (6, 7). The appearance of androgen receptor splice variants (AR-Vs) is thought to play a role in the resistance to treatment (8).

AR-Vs, which lack the C-terminal ligand-binding domain but retain the transactivating N-terminal domain, are constitutively activated as transcription factors and activate the target genes without any ligand (9, 10). In various AR-Vs, androgen receptor splice variant 7 (AR-V7) encodes a functional protein and is detectable in clinical simples (11). Some studies have demonstrated an association between AR-V7 status and the treatment outcomes of prostate cancer (12, 13). However, the prognostic value of AR-V7 status for the treatment response of mCRPC remains unclear. Although the expression of AR-V7 has been shown to be negatively correlated with the efficacy of enzalutamide and abiraterone in several clinical studies, there are still studies to that report the opposite findings (14). In addition, some studies have investigated the role of AR-V7 in mCRPC patients who received chemotherapy, but the sample sizes of most studies were limited, so the prognostic value of AR-V7 in the personalized treatment of mCRPC was restricted (15, 16).

Therefore, we conducted this meta-analysis and summarized the available data from clinical studies to assess the prognostic value of AR-V7 in the treatment outcomes of mCRPC. The primary aim of our meta-analysis was to compare the prostate-specific antigen (PSA) response, progression-free survival (PFS), and overall survival (OS) of mCRPC patients with different AR-V7 statuses treated with ARSis or taxanes. We also performed subgroup analysis according to the detection methods for AR-V7 status.

## Materials and Methods

This work was executed in accordance with the Preferred Reporting Items for Systemic Reviews and Meta-analysis (PRISMA) guidelines (12). It was also registered in the International Prospective Register of Systematic Reviews (PROSPERO) before screening studies for inclusion began (CRD42020152263).

### Search Strategy and Selection Criteria

We conducted a systematic literature search by searching the PubMed, Embase, and MEDLINE databases. Studies that assessed the association between AR-V7 status and treatment outcomes of patients with mCRPC and that were published between January 1974 and September 2019 retrieved. The complete retrieval strategy was: (((((AR-V7) OR AR3) OR androgen receptor splicing variants 7) OR androgen receptor 3)) AND ((((prostate cancer) OR prostate tumor) OR prostate neoplasm) OR prostate carcinoma). In addition, the reference lists of the included studies and related reviews and reports were screened.

The inclusion criteria were as follows. (1) Studies reported the association between AR-V7 status at baseline and time-to-events outcomes for mCRPC patients treated with ARSis or chemotherapy, including PSA response, clinical and/or radiographic PFS or OS. (2) Odds ratios (ORs) or hazard ratios (HRs) with corresponding 95% confidence intervals (CIs) were reported directly or could be calculated. (3) The clinical studies were performed with adults and were published in English. The exclusion criteria were as follows. (1) The study was a review, case report, comment, editorial, or meta-analysis. (2) The study only contained an AR-V7-positive or an AR-V7-negative cohort but not boths. (3) The Study only reported the Kaplan-Meier survival curve without available HRs and 95% CI. If there were multiple reports published as sequential studies from the same cohort, the most recent follow-up report was included.

According to the inclusion and exclusion criteria, the initial selection of studies was based on the titles and abstracts of all studies. Then we assessed the full texts of the potential studies. All studies were independently screened by two investigators (JW and YZ). A third researcher (CW) was consulted to resolve disagreements.

### Data Extraction and Quality Assessment

Data extraction was conducted by two reviewers (XG and PY) independently. Basic information and patient characteristics were extracted from all included studies. To analyze the potential predictive value of AR-V7, PSA response rates and the HRs (95% CIs) of PFS and OS in patients with different AR-V7 statuses and different treatment regimens were collected. The Newcastle-Ottawa Quality Assessment Scale (NOS) was applied to assess the quality of the eligible studies by two trained investigators (JG and RL). Discrepancies were resolved by discussion with a third reviewer (ZL).

### Statistical Analysis

According to the classification criteria reported in the included articles, patients were divided into two groups, AR-V7 positive and negative. ORs with 95% CIs were used to analyze the correlation between AR-V7 status and PSA response. HRs with 95% CIs were used to estimate the association between AR-V7 status and PFS and OS. We also performed a subgroup analysis based on the methods for the detection of AR-V7.

The meta-analysis was performed by using Stata software (version 12.0; College station, TX, USA). *P* < 0.05 was considered statistically significant. Considering the relatively conservative results, we used random effect models for this meta-analysis. The heterogeneity was evaluated by the *I^2^* statistic. Publication bias was detected by Begg’s funnel plot and Egger’s regression test if the number of included studies was more than ten. Publication bias was considered to be significant if Begg’s funnel plot was asymmetric or if *P* < 0.10 for Egger’s test. For a meta-analysis with more than ten studies, sensitivity analysis was applied to verify the stability of the results.

## Results

### Characteristics of the Included Studies

**Figure 1:**
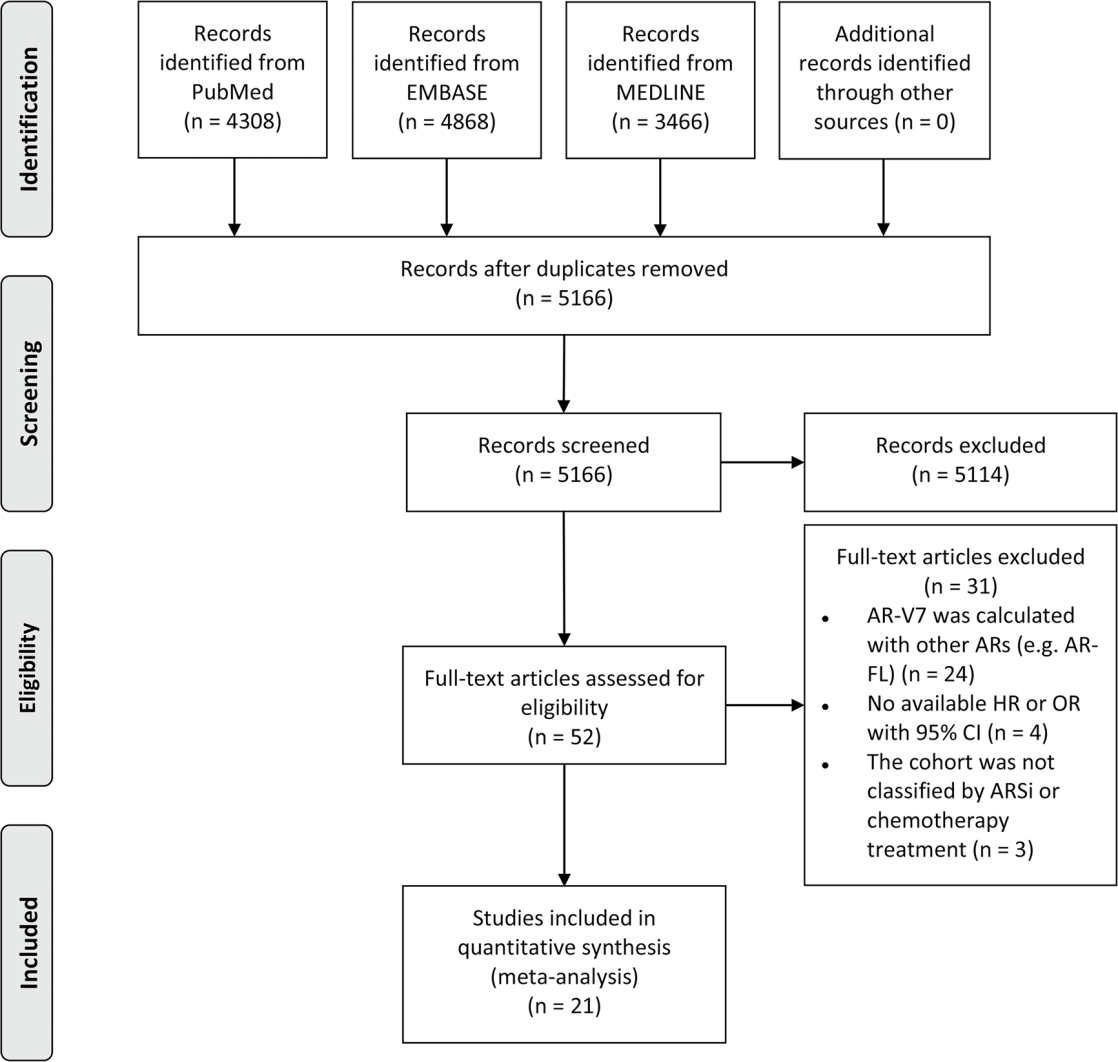
PRISMA flow diagram of literature screening. AR-V7, androgen receptor splice variant 7; AR, androgen receptor; AR-FL, full-length androgen receptor; HR, hazard ratio; OR, odds ratio; CI, confidence interval; ARSi, androgen receptor signaling inhibitor.

The flow diagram of the search and screening process is shown in **Figure 1**. A total of 21 studies with 1,545 patients were included in this meta-analysis; 16 studies reported the prognostic value of AR-V7 status in predicting the treatment outcomes of the mCRPC patients treated with ARSis (including enzalutamide and abiraterone), and 8 studies reported the prognostic value of AR-V7 status for predicting the treatment outcomes of the mCRPC patients treated with chemotherapy (taxane). The therapeutic effect of ARSis and chemotherapy in mCRPC patients with different AR-V7 statuses was compared in 3 studies. The characteristics of the clinical features of the included patients are shown in **Table 1**. The definition of PSA response, PFS, OS, and details of the AR-V7 detection assay in the included studies are described in the **Table S1** and **S2** separately.

**Table 1:** Characteristics of included studies and patients.

Study	Year	Region	Study design	AR-V7 detection method	Patients’ characteristics						
					Patients (n)	Age, median (range), years	No. (%) GS≧8	PSA, median (range), ng/mL	ALP, median (range), U/L	Treatment	Median follow-up, median (range), months
Antonarakis^a^	2017	USA	Prospective	RT-PCR	36	70^b^	30 (83)	92.0^b^	120^b^	E or A	14.6^b^
					113	71^b^	68 (60)	31.4^b^	96^b^	E or A	21.7^b^
Antonarakis	2015	USA	Prospective	RT-PCR	37	67 (46–82)	29 (83)	126 (0.1–2,270)	161 (53–1,243)	Taxane	7.7 (0.7–19.0)
Antonarakis	2014	USA	Prospective	RT-PCR	31	69 (48–79)	22 (73)	37.8(2.2–2,045.0)	118 (59–1,348)	A	4.6 (0.9–8.2)
					31	70 (56–84)	18 (60)	44.3 (4.3–3,204.2)	108 (58–872)	E	5.4 (1.4–9.9)
Armstrong	2019	USA	Prospective	RT-PCR and IFS	118	73 (45–92)	68 (58)	19 (0.08–4,194)	NA	E or A	19.6^b^
Del Re	2016	Italy	Prospective	dd-PCR	36	66 (51–81)	19 (53)	26.3 (0.63–4,581)	180 (49–917)	E or A	9.0 (2.0–31.0)
Nakazawa	2015	USA	Prospective	RT-PCR	12	65 (50–82)	13 (92)	54.7 (2.2–895)	112 (53–838)	E or A or taxane	11.0 (6.0–18.0)
Okegawa^a^	2018	Japan	Retrospective	RT-PCR	26	72^b^	25 (96)	79.1^b^	378^b^	E or A	NA
					23	71^b^	21 (91)	71.5^b^	323^b^	E or A	NA
Onstenk	2015	Netherlands	Prospective	RT-PCR	29	70 (7)^c^	NA	321 (76–649)	163 (106–375)	C	7.0 (2.0–27.0)
Qu	2016	USA	Retrospective	dd-PCR	81	68 (46–89)	40 (49)	16.4 (0.1–972.1)	NA	A	NA
					51	69 (50–88)	21 (41)	45.5 (0.3–1,148.4)	NA	E	NA
Scher	2018	UK/USA	Prospective	IFS	142	70 (40–91)	NA	68.3 (0.05–16,275)	112 (44–1,055)	E or A or taxane	NA
Scher	2016	USA	Prospective	IFS	130	68 (45–87)	NA	28.0 (0.1–2,454.5)	99 (25–2,170)	E or A	NA
					63	68 (48–91)	NA	99.5 (0.1–3,728.2)	181 (49–1,816)	Taxane	NA
Seitz	2017	Germany	Prospective	dd-PCR	85	71 (66–74)^d^	NA	211 (29–768)^d^	NA	E or A	7.6 (4.7–12.7)^d^
Sharp	2019	UK/USA	Retrospective	IHC	160	68 (64–73)^d^	NA	230.5 (77.0–591.5)^d^	127.0 (72.3–332.5)^d^	E or A or taxane	NA
Sieuwerts^a^	2019	Netherlands	Prospective	RT-PCR	25	68 (7)^c^	NA	232 (77–707)^d^	192 (127–310)^d^	C	NA
					27	70 (8)^c^	NA	186 (64–500)^d^	150 (68–368)^d^	C	NA
Steinestel^a^	2015	Germany	Prospective	RT-PCR	18	74 (53–82)	11 (65)	239.9 (13.9–4,282)	NA	E or A or D	NA
					19	72 (56–87)	11 (58)	88.6 (0.1–1,374)	NA	E or A or D	NA
Tagawa	2018	USA	Prospective	dd-PCR	54	71 (53–84)	31 (61)	92.1 (2.4–1,558.0)	217.8 (260.35)^c^	Taxane	NA
Takeuchi	2016	Japan	Retrospective	Nested PCR	43	73 (59–88)	31 (72)	130 (5.3–9,529)	NA	E or A	NA
To^a^	2018	Australia	Prospective	RT-PCR	9	77 (46–89)	5 (56)	49.2 (3–703)	NA	E or A	NA
					28	75.5 (52–89)	13 (46)	42.5 (0.8–588)	NA	E or A	NA
Todenhöfer	2016	Canada	Prospective	RT-PCR	37	70 (53–87)	NA	NA	116 (45–1,869)	A	NA
Welti	2016	UK	Retrospective	IHC	37	67.5 (64.2–75.3)^d^	NA	NA	142.0 (69.5–448.5)^d^	E or A or taxane	NA
Zhu^e^ (JHU cohort)	2017	USA	Retrospective	RISH	28	64 (52–86)	22 (79)	59.6 (0.7–6,746.8)	NA	E or A	NA
Zhu^e^ (UK cohort)	2017	UK	Retrospective	RISH	16	72.3 (48.8–79.4)	12 (75)	177.0 (2.6–4,098.0)	NA	E or A	NA

^a^The characteristics of AR-V7-positive patients were described on the upper sub-row and the characteristics of AR-V7-negative patients were described on the lower sub-row.

^b^Range was absent.

^c^Mean (standard deviation, SD).

^d^Interquartile range, IQR.

^e^JHU and UK were the 2 cohorts described respectively by Zhu et al.

AR-V7, androgen receptor splice variant 7; GS, Gleason score; PSA, prostate-specific antigen; ALP, alkaline phosphatase; RT-PCR, reverse-transcription polymerase chain reaction; E, enzalutamide; AA, abiraterone acetate; IFS, immunofluorescent staining; NA, not applicable; dd-PCR, droplet-digital polymerase chain reaction; C, cabazitaxel; D, docetaxel; IHC, immunohistochemistry; PCR, polymerase chain reaction; RISH, RNA in situ hybridization.

### The Association Between AR-V7 Expression Status and Outcomes in mCRPC Patients Treated With ARSis

**Figure 2:**
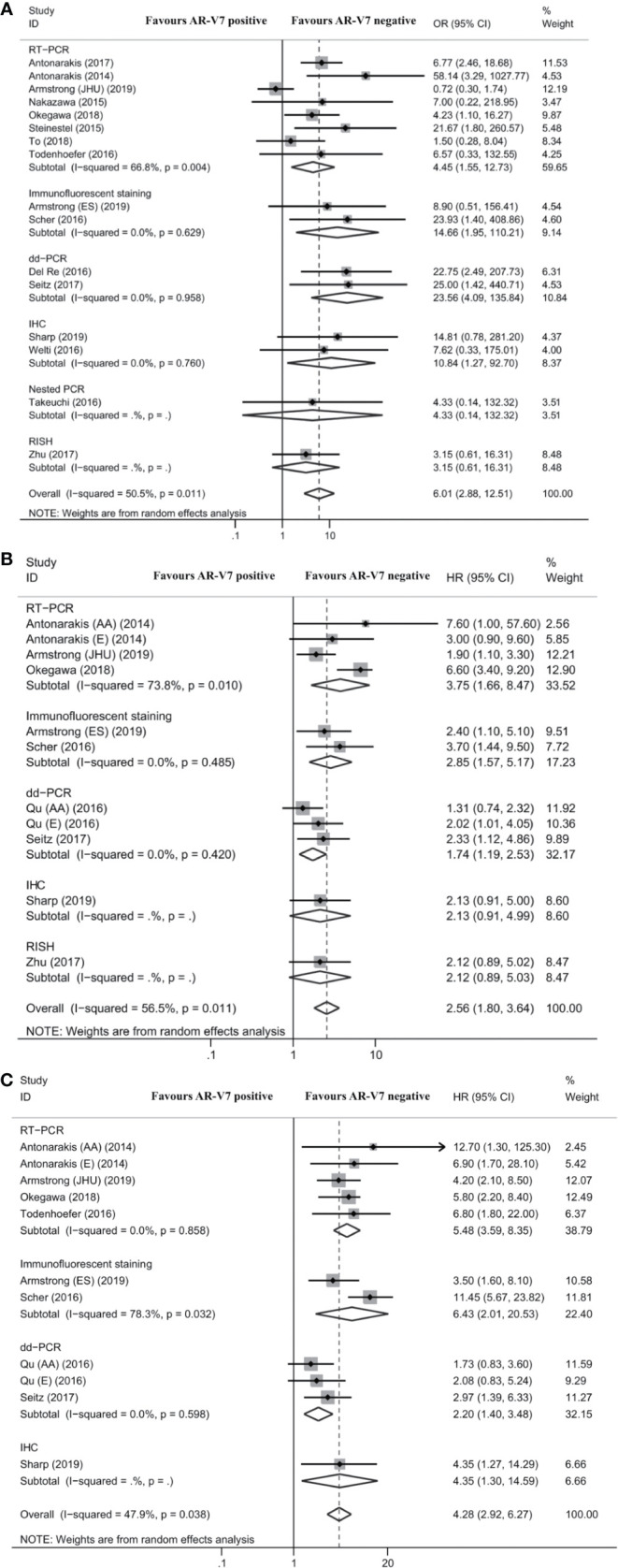
Forest plot of mCRPC patients treated with androgen receptor signaling inhibitors by different AR-V7 status. **(A)** prostate-specific antigen (PSA) response rate; **(B)** progression free survival (PFS); and **(C)** overall survival (OS). JHU and ES were the 2 cohorts described respectively by Armstrong et al. AR-V7, androgen receptor splice variant 7; OR, odds ratio; CI, confidence interval; RT-PCR, reverse transcriptase-polymerase chain reaction; dd-PCR, digital-droplet polymerase chain reaction; IHC, immunohistochemistry; PCR, polymerase chain reaction; RISH, RNA in situ hybridization; HR, hazard ratio; AA, abiraterone acetate; E, enzalutamide.

Fifteen studies reported the association between AR-V7 status and PSA response in mCRPC patients treated with ARSis. The overall proportion of AR-V7-positive patients with PSA response was 19.3% (95% CI 14.6–24.9%), while the PSA response rate of AR-V7-negative patients was 45.7% (95% CI 41.8–49.5%; **Table S3**). For mCRPC patients treated with ARSis, AR-V7-positive patients had a lower PSA response rate than AR-V7-negative patients (OR 6.01, 95% CI 2.88–12.51; *P* < 0.001; **Figure 2A**). There was significant heterogeneity among these studies (*I^2^* = 50.5%; *P* = 0.011). Subgroup analysis revealed that in the studies that used reverse transcription-polymerase chain reaction (RT-PCR) (OR 4.45, 95% CI 1.55–12.73; *P* = 0.005), immunofluorescent staining (IFS) (OR 14.66, 95% CI 1.95–110.21; *P* = 0.009), droplet-digital PCR (dd-PCR) (OR 23.56, 95% CI 4.09–135.84; *P* < 0.001), or immunohistochemistry (IHC) (OR 10.84, 95% CI 1.27–92.70; *P* = 0.029) for the detection of AR-V7, there were statistically significant differences in PSA response rates between AR-V7-positive and AR-V7-negative patients (**Figure 2A**).

Eight studies reported the association between AR-V7 status and PFS of mCRPC patients treated with ARSis. As shown in **Figure 2B**, PFS was significantly shorter among AR-V7-positive patients than AR-V7-negative patients (HR 2.56, 95% CI 1.80–3.64; *P* < 0.001). There was significant heterogeneity among these studies (*I^2^* = 56.5%; *P* = 0.011). Subgroup analysis revealed that in the studies that used RT-PCR (HR 3.75, 95% CI 1.66–8.47; *P* = 0.001), IFS (HR 2.85, 95% CI 1.57–5.17; *P* = 0.001), and dd-PCR (HR 1.74, 95% CI 1.19–2.53; *P* = 0.004) for the detection of AR-V7, AR-V7-positive patients had significantly shorter PFS than AR-V7-negative patients (**Figure 2B**).

Eight studies reported the association between AR-V7 status and OS of mCRPC patients treated with ARSis. OS was significantly shorter in AR-V7-positive patients than in AR-V7-negative patients (HR 4.28, 95% CI 2.92–6.27; *P* < 0.001; **Figure 2C**). There was significant heterogeneity among these studies (*I^2^* = 47.9%; *P* = 0.038). Subgroup analysis revealed that in the studies that used RT-PCR (HR 5.48, 95% CI 3.59–8.35; *P* < 0.001), IFS (HR 6.43, 95% CI 2.01–20.53; *P* = 0.002), and dd-PCR (HR 2.20, 95% CI 1.40–3.48; *P* = 0.001) for the detection of AR-V7, AR-V7-positive patients had significantly shorter OS than AR-V7-negative patients (**Figure 2C**).

### The Association Between AR-V7 Expression Status and Outcomes in mCRPC Patients Treated With Chemotherapy

**Figure 3:**
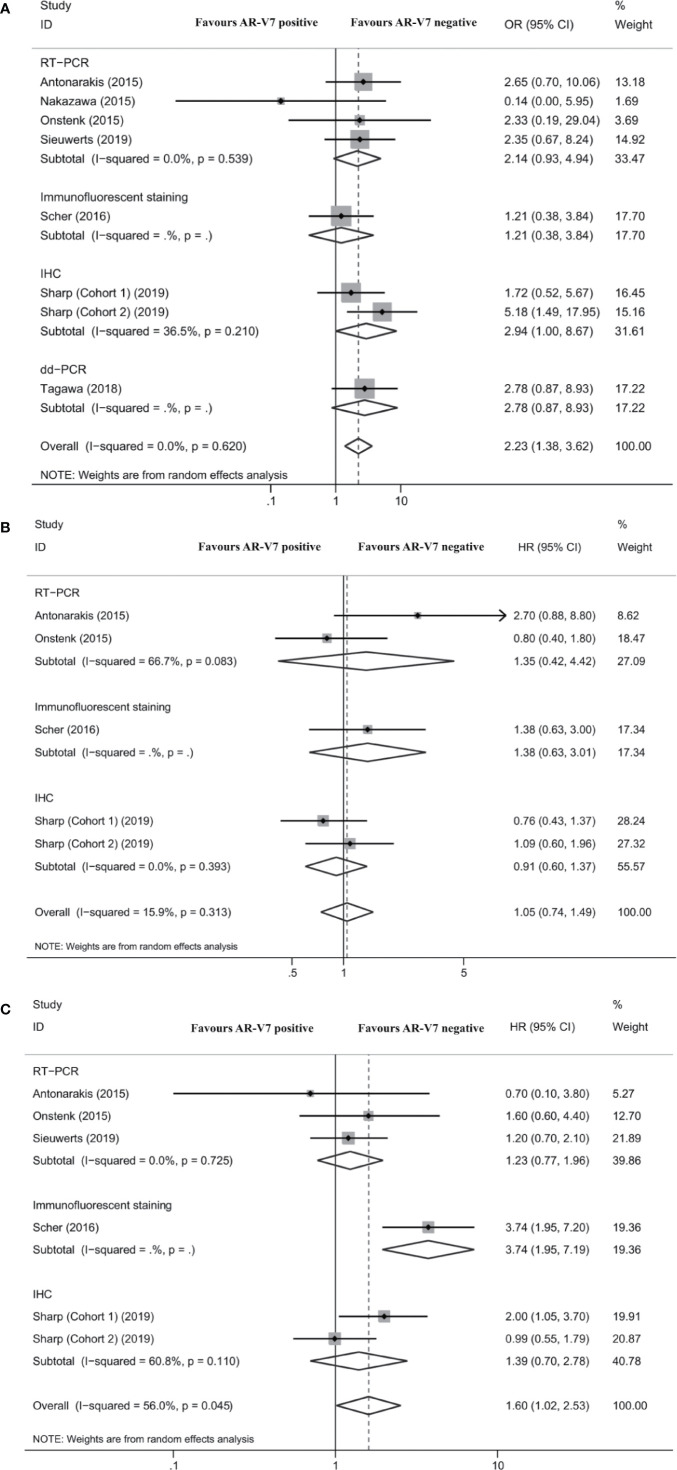
Forest plot of mCRPC patients treated with chemotherapy with different AR-V7 status. **(A)** prostate-specific antigen (PSA) response rate; **(B)** progression free survival (PFS); and **(C)** overall survival (OS). AR-V7, androgen receptor splice variant 7; OR, odds ratio; CI, confidence interval; RT-PCR, reverse transcriptase-polymerase chain reaction; IHC, immunohistochemistry; dd-PCR, digital-droplet polymerase chain reaction; HR, hazard ratio.

Seven studies reported the association between AR-V7 status and PSA response in mCRPC patients treated with chemotherapy. The PSA response rate of AR-V7-positive patients was 28.2% (95% CI 22.0–35.3%), while the PSA response rate of AR-V7-negative patients was 44.1% (95% CI 36.4–52.1%; **Table S4**). Although each subgroup did not report obvious differences, the total difference was statistically significant (OR 2.23, 95% CI 1.38–3.62; *P* = 0.001; **Figure 3A**). No significant heterogeneity was detected among these studies (*I^2^* = 0.0%; *P* = 0.620). The differences were also nonsignificant in the subgroup analysis based on AR-V7 detection methods (**Figure 3A**).

Four studies reported the association between AR-V7 status and PFS of mCRPC patients treated with chemotherapy. The difference of PFS between patients with different AR-V7 statuses was nonsignificant (HR 1.05, 95% CI 0.74–1.49; *P* = 0.796; **Figure 3B**). No significant heterogeneity was detected among these studies (*I^2^* = 15.9%; *P* = 0.313; **Figure 3B**).

Five studies reported the association between AR-V7 status and OS in mCRPC treated with chemotherapy. AR-V7-positive patients had a significantly shorter OS than AR-V7-negative patients (HR 1.60, 95% CI 1.02–2.53; *P* = 0.043; **Figure 3C**). There was significant heterogeneity among these studies (*I^2^* = 56.0%; *P* = 0.045). Subgroup analysis revealed that in the studies that used IFS, there was a statistically significant difference in OS between AR-V7-positive and AR-V7-negative patients (HR 3.74, 95% CI 1.95–7.19; *P* < 0.001; **Figure 3C**).

### The Differences in OS in mCRPC Patients Receiving ARSis or Chemotherapy

**Figure 4:**
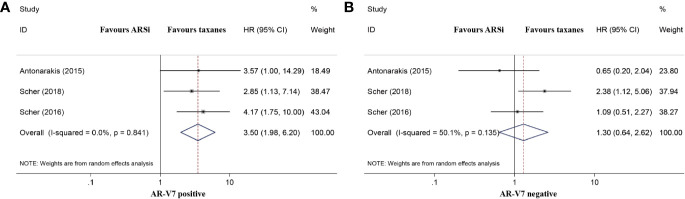
Forest plot of overall survival (OS) of mCRPC patients treated with androgen receptor signaling inhibitiors (ARSi) or chemotherapy. **(A)** For AR-V7 positive patients, those receiving ARSi had a shorter median OS than those receiving chemotherapy; **(B)** For AR-V7 negative patients, there was no significant difference for the median OS between mCRPC patients treated with ARSi and chemotherapy. ARSi, androgen receptor signaling inhibitors; HR, hazard ratio; CI, confidence interval; AR-V7, androgen receptor splice variant 7.

Three studies compared the OS of mCRPC patients treated with chemotherapy or ARSis. For AR-V7-positive patients, those receiving ARSis had shorter OS than those receiving chemotherapy (HR 3.50, 95% CI 1.98–6.20; *P* < 0.001; **Figure 4A**). However, the difference in OS between AR-V7-negative mCRPC patients treated with ARSis and chemotherapy was nonsignificant (HR 1.30, 95% CI 0.64–2.62; *P* = 0.471; **Figure 4B**). No significant heterogeneity was detected among these studies (*I^2^* = 0.0%, *P* = 0.841 for AR-V7 positive; *I^2^* = 50.1%, *P* = 0.135 for AR-V7 negative).

### Quality Assessment, Sensitivity Analysis, and Publication Bias

According to the NOS, all included studies were judged to be of intermediate or high quality (**Table S5**). Additionally, no obvious bias was found according the sensitivity analysis of the studies that examined the value of using AR-V7 status to predict PSA response of patients with mCRPC treated with ARSis (**Figure S1**).

Begg’s test and Egger’s test were utilized to assess the publication bias of the studies examining the prognostic value of AR-V7 for predicting the outcomes of mCRPC patients with ARSis, including PSA response, PFS and OS (**Figure S2-S4**). The results of both Begg’s test (**Figure S2A**) and Egger’s test (PSA response: *P*_Egger’s_ test = 0.005; **Figure S2B**) revealed publication bias in the studies that examined PSA response as the outcome. No obvious publication bias was found among the studies that examined PFS and OS as outcomes using Egger’s test (PFS: *P*_Egger’s_ test = 0.945; OS: *P*_Egger’s_ test = 0.596). In addition, no obvious asymmetry was found in Begg’s funnel plot for the studies that examined PFS and OS (**Figure S3A** and **S4A**).

## Discussion

With the use of ARSis and chemotherapy, patients with mCRPC experienced a significant increase in survival (17–19). Although these treatments are able to control the development of mCRPC for a few months to about 24 months, the benefits of ARSis and chemotherapy are still not long lasting (20). With an increase in treatment time, the gradually serious drug resistance reminds us to pay attention to the disease. In 2014, Antonarakis et al. demonstrated that AR-V7 was a potential biomarker of treatment outcomes for mCRPC patients treated with abiraterone and enzalutamide (12). Since then, it has been unclear whether AR-V7 could be used as a predictive molecule for ARSis and chemotherapy in mCRPC patients.

Our meta-analysis suggests that AR-V7 is a biomarker for the application of ARSis in patients with mCRPC. That is, AR-V7-positive mCRPC patients treated with ARSis had poorer PSA response and shorter PFS and OS than AR-V7-negative patients. Among the outcome events, the differences in the PSA response rate between AR-V7-positive and AR-V7-negative mCRPC patients could be found by using RT-PCR, IFS, dd-PCR, and IHC but not nested polymerase chain reaction (PCR) or RNA *in situ* hybridization (RISH), which might be due to the limited sample sizes of the studies that used nested PCR or RISH. Takeuchi et al. (21) reported that they produced 30 cycles of nested PCR by substituting each corresponding antisense primer after 35 cycles of PCR, which could improve the detection rate of AR-V7. Zhu et al. (22) conducted a novel RISH assay and quantitative analysis of AR-V7 mRNA levels in formalin−fixed, paraffin−embedded (FFPE) biopsies from mCRPC patients. They also proved that the IHC results are robustly associated with the RISH results. More research is needed to certify the sensitivity and specificity of nested PCR and RISH. PFS and OS were also assessed to compare the prognosis of mCRPC patients treated with ARSis in disparate AR-V7 status. In subgroup analysis, AR-V7-negative mCRPC patients treated with ARSis had longer PFS and OS than AR-V7- negative patients by using RT-PCR, IFS, and dd-PCR but not IHC and RISH, which might be due to the heterogeneity in the characteristics of the included studies and the sensitivity and specificity of different AR-V7 detection methods. It is worth mentioning that, although advanced and aggressive prostate cancer tissue may have a higher AR-V7 expression (23), several studies have proved that AR-V7 was an independent predictive factor for CRPC prognosis through multivariate analysis (12, 24). In addition, AR-V7 expression may be a reflection of prior exposure to anti-androgen therapy (25). Therefore, considering the existing research, we consider AR-V7 to be a predictive biomarker for mCRPC patients treated with ARSis, and AR-V7-negative patients have better treatment outcomes. What’s more, the detection of AR-V7 before treatment for mCRPC patients is important for the selection of treatment regimens, even though the AR-V7 expression has been tested during the period of HSPC.

However, the prognostic value of AR-V7 in the treatment of mCRPC with taxane has yet to be elucidated (26). In our meta-analysis, AR-V7-positive mCRPC patients treated with taxane had a worse PSA response and shorter OS than AR-V7-negative patients, but PFS did not differ much between patients with different AR-V7 statuses. None of the subgroup showed that AR-V7-negative patients treated with chemotherapy had a greater PSA response, which meant that the results lacked stability. The prognostic value of AR-V7 status for the chemotherapy outcomes of mCRPC needs to be evaluated in more clinical studies. In addition, we also compared the OS of mCRPC patients treated with ARSis and chemotherapy with different AR-V7 statuses. The result suggests that AR-V7-positive patients would be better treated with taxane than ARSis. However, for AR-V7-negative mCRPC patients, there was no significant difference in OS between ARSis or taxane. Interestingly, in 2016, a phase 1b study (COU-AA-206) determined the safe dose combination of docetaxel and abiraterone acetate plus prednisone (27), which might be a promising choice for AR-V7-positive patients. However, further studies on validating the efficacy and safety of this treatment option are needed.

The advantages and disadvantages of AR-V7 detection methods were discussed before (28). Among AR-V7 detection methods, RT-PCR-based tests are the most widely used (12, 15, 16, 20, 25, 29–34). This method is highly sensitive, but its clinical application may be limited because of a defective reliance on the detection of circulating tumor cells (CTCs) and low levels of analytes in liquid biopsy samples. Nested PCR and dd-PCR are improved methods based on PCR. In the former, a complete fragment is amplified using two pairs of PCR primers instead of one pair in nested PCR, which improves the specificity of the results (21). dd-PCR can be used for the absolute quantification of the transcript without the need for normalization or external reference genes (35). IHC and IFS could provide an in-situ visualization of protein, but the development of an optimized antibody is technically challenging and time-consuming (22). Fortunately, novel specific antibodies have been reported to detect the expression of AR-V7 in tissues (23, 36). RISH, which is based on FFPE or fresh frozen mCRPC specimens and avoids CTC detection, could make an in-situ visualization of mRNA, but pre−mRNA is sometimes detected (28). Details of the target samples and AR-V7 detection methods used in the included studies are listed in **Table S2**.

Considering the heterogeneity among the included studies, we also analyzed the possible risk of bias and the potential limitations of the studies. First, only observational studies were included in our meta-analysis and some clinical characteristics such as visceral disease and pain status were not considered. Second, the differences in patients’ baseline characteristics may influence the pooled results. The Gleason score was only reported in 13 studies and the proportion of Gleason scores greater than 8 points was also inconsistent in the included studies (12, 15, 16, 20, 22, 25, 29, 30, 32, 33, 37–39). Therefore, the differences in patients’ baseline information may influence the pooled results. Third, the included studies did not limit the previous treatment for mCRPC patients, thus inhibiting us from ruling out the delayed effects of previous treatments. In addition, the mechanisms of abiraterone and enzalutamide were not the same (35, 40, 41). It is difficult to evaluate the therapeutic effects of abiraterone and enzalutamide separately in the 12 studies (2, 13, 20–23, 25, 29, 30, 33, 36, 37, 42). Fourth, the differences in AR-V7 detection methods among different studies was a source of bias. However, in the subgroup analysis, the predictive effect of AR-V7 on the treatment outcomes of mCRPC patients treated with ARSis or chemotherapy could be evaluated based on different assays. Definite differences might exist during actual operations in separate laboratories. Currently, there was no recognized gold standard for AR-V7 detection. More diagnostic tests should be used to compare the sensitivity and specificity of various AR-V7 detection methods.

## Conclusion

In summary, AR-V7 is a potential biomarker of treatment resistance in mCRPC patients and AR-V7-positive mCRPC patients had shorter OS than AR-V7-negative patients when treated with ARSi or taxane. AR-V7-positive mCRPC patients showed poorer outcomes including PSA response, PFS, and OS than AR-V7-negative patients when receiving ARSis. AR-V7-positive patients had better outcomes when treated with taxane than ARSis. The detection methods for AR-V7 may influence the prognostic value of AR-V7 status on the treatment outcome. The detection of AR-V7 before treatment for mCRPC patients is important for the selection of treatment regimens.

## Data Availability Statement

All datasets presented in this study are included in the article/**Supplementary Material**.

## Author Contributions

Conceptualization: SW and XL. Methodology: YZ. Investigation: XG and PY. Data curation: JG and RL. Formal analysis: JW, CW, and ZL. Writing, review and editing of the manuscript: JW, YZ, TW, and XL. Study supervision: JL and XL. All authors contributed to the article and approved the submitted version.

## Funding

This work was supported by Innovation Foundation of Huazhong University of Science and Technology (Grant Number 2019kfyXKJC06; principal investigator XL) and National Natural Science Foundation of China (Grant Number: 81702518 and 81500636; principal investigator XL).

## Conflict of Interest

The authors declare that the research was conducted in the absence of any commercial or financial relationships that could be construed as a potential conflict of interest.
