# Supplementary material for: Prognostic Value of Androgen Receptor Splice Variant 7 in the Treatment of Metastatic Castration-Resistant Prostate Cancer: A Systematic Review and Meta-Analysis
Source: Front Oncol. 2020 Nov 30;10:562504. doi: 10.3389/fonc.2020.562504 (PMC7735106; doi:10.3389/fonc.2020.562504)
Supplement: Supplementary file 1 [file DataSheet_1.docx]

**Prognostic value of androgen receptor splice variant 7 in the treatment of metastatic castration-resistant prostate cancer: A systematic review and meta-analysis**

**Supplementary materials**

**Table of Contents**

|  |  | **Page** |
| --- | --- | --- |
| **Part 1** | PICOS components | **3** |
| **Part 2** | The Newcastle-Ottawa Quality Assessment Scale (NOS) | **3-4** |
| **Part 3** | Supplemental tables | **5-13** |
| **Table S1** | Definition of PSA response, PFS and OS in the studies included in the meta-analysis | **5-7** |
| **Table S2** | Target simples and AR-V7 detection method of the included studies | **8-10** |
| **Table S3** | PSA response rate of mCRPC patients treated with ARSi in different AR-V7 status | **11** |
| **Table S4** | PSA response rate of mCRPC patients treated with taxane in different AR-V7 status | **12** |
| **Table S5** | Quality assessment of included studies | **13** |
| **Part 4** | Supplemental figures | **14-17** |
| **Figure S1** | Sensitivity analysis of AR-V7 status in predicting PSA response of the mCRPC patients treated with ARSi. | **14** |
| **Figure S2** | Publication bias of studies the reported PSA response of mCRPC patients treated with ARSi. | **15** |
| **Figure S3** | Publication bias of studies the reported PFS of mCRPC patients treated with ARSi. (A) Begg’s funnel plot; (B) Egger’s linear regression plot. | **16** |
| **Figure S4** | Publication bias of studies the reported OS of mCRPC patients treated with ARSi. | **17** |
| **Part 5** | References | **18-19** |

**Part 1 PICOS components**

We have designed four research programs

1. AR-V7 status in predicting the prognosis of the patients with mCRPC treated with ARSi.

Patients: Patients suffered from mCRPC and treated with ARSi

Exposure: AR-V7 positive

Comparison: AR-V7 negative

Outcomes: Prostate-specific antigen (PSA) response rate, progression-free survival (PFS), and overall survival (OS)

Study design: Prospective cohort or retrospective cohort study

2. AR-V7 status in predicting the prognosis of the patients with mCRPC treated with chemotherapy (taxane).

Patients: Patients suffered from mCRPC and treated with chemotherapy (taxane)

Exposure: AR-V7 positive

Comparison: AR-V7 negative

Outcomes: PSA response rate, PFS, and OS

Study design: Prospective cohort or retrospective cohort study

3. Compare the therapeutic effect of ARSi and chemotherapy in AR-V7-positive patients.

Patients: Patients suffered from mCRPC with AR-V7 positive

Exposure: treated with ARSi

Comparison: treated with chemotherapy

Outcomes: OS

Study design: Randomized clinical trial, prospective cohort or retrospective cohort study

4. Compare the therapeutic effect of ARSi and chemotherapy in AR-V7-negative patients.

Patients: Patients suffered from mCRPC with AR-V7 negative

Exposure: treated with ARSi

Comparison: treated with chemotherapy

Outcomes: OS

Study design: Randomized clinical trial, prospective cohort or retrospective cohort study

**Part 2 The Newcastle-Ottawa Quality Assessment Scale (NOS)**

A study can be awarded a maximum of one star for each numbered item within the Selection and Outcome categories. A maximum of two stars can be given for Comparability. A study can be scored a maximum of 4 star for selection, 2 stars for comparability and 3 stars for outcome. The scores ranging from 0 to 3 were considered as lower quality, scores ranging from 4 to 5 were considered as moderate quality, and scores ranging from 6 to 9 were considered as high quality.

Details of NOS were shown below.

**Selection**

1) Representativeness of the exposed cohort

a) truly representative of the average _______________ (describe) in the community*

b) somewhat representative of the average ______________ in the community*

c) selected group of users e.g. nurses, volunteers

d) no description of the derivation of the cohort

2) Selection of the non-exposed cohort

a) drawn from the same community as the exposed cohort*

b) drawn from a different source

c) no description of the derivation of the non-exposed cohort*

3) Ascertainment of exposure

a) secure record (e.g. surgical records) *

b) structured interview*

c) written self-report

d) no description

4) Demonstration that outcome of interest was not present at start of study

a) yes *

b) no

**Comparability**

1) Comparability of cohorts on the basis of the design or analysis

a) study controls for _____________ (select the most important factor) *

b) study controls for any additional factor* (These criteria could be modified to indicate specific

control for a second important factor.)

**Outcome**

1) Assessment of outcome

a) independent blind assessment *

b) record linkage *

c) self-report

d) no description

2) Was follow-up long enough for outcomes to occur

a) yes (select an adequate follow up period for outcome of interest) *

b) no

3) Adequacy of follow up of cohorts

a) complete follow up - all subjects accounted for *

b) subjects lost to follow up unlikely to introduce bias - small number lost - > ____ % (select an

adequate %) follow up, or description provided of those lost) *

c) follow up rate < ____% (select an adequate %) and no description of those lost

d) no statement

**Part 3 Supplementary tables**

**Table S1. Definition of PSA response, PFS and OS in the studies included in the meta-analysis**

| **Study** | **Year** | **Definition** | | |
| --- | --- | --- | --- | --- |
|  |  | **PSA response** | **PFS** | **OS** |
| Antonarakis ^1^ | 2017 | patients with a ≥ 50% PSA decline from baseline at any time point after therapy (and maintained for ≥ 3 weeks) | not mentioned | the interval from enrolment to death from any cause |
| Antonarakis ^2^ | 2015 | patients who achieved at least a 50% PSA level decline from baseline at any time point after therapy (and maintained it for ≥ 3 weeks) | symptomatic progression (worsening disease-related symptoms or new cancer-related complications), radiologic progression (on CT scan, ≥20% enlargement in sum diameter of soft-tissue target lesions RECIST; on bone scan, ≥2 new bone lesions), or death, whichever occurred first. | the time to death from any cause. |
| Antonarakis ^3^ | 2014 | ≥50% decline in PSA level from baseline, maintained for ≥4 weeks | symptomatic progression (worsening disease-related symptoms or new cancer-related complications), radio-graphic progression (≥20% increase in the sum of the diameters of soft-tissue target lesions on CT scanning (according to the RECIST) or ≥2 new bone lesions on bone scanning), or death, whichever occurred first. | the time to death from any cause |
| Armstrong ^4^ | 2019 | PSA declines ≥50% were confirmed with a subsequent PSA value 2 or more weeks later | date of registration to clinical/radiographic progression or death, whichever occurred first. Radiographic progression was assessed using PCWG2 soft tissue and bone scan criteria. Clinical progression was defined by death, pain, or other symptomatic progression; initiation of new systemic therapy; or a skeletal-related event. | the time to death from any cause |
| Del Re ^5^ | 2016 | 50% reduction in PSA level from baseline | not mentioned | not mentioned |
| Nakazawa ^6^ | 2015 | ≥50% PSA decline at any time on therapy, maintained for ≥4 weeks | not mentioned | not mentioned |
| Okegawa ^7^ | 2018 | a 50% or greater decline from baseline at 12 weeks | independent blinded review of available radionuclide bone scans, CT, or MRI, using the PCWG2 criteria (rPFS was defined as ≥2 new lesions on an 8-week bone scan plus two additional lesions on a confirmatory scan, ≥2 new confirmed lesions on any scan ≥12 weeks after random assignment, progression in nodes or viscera on cross-sectional imaging, or death | not mentioned |
| Onstenk ^8^ | 2015 | 50% decline in PSA level from baseline to 12 weeks | interval between registration and progression of disease or death | interval between registration and death |
| Qu ^9^ | 2016 | not mentioned | time to treatment failure | the time to death from any cause |
| Scher ^10^ | 2018 | not mentioned | not mentioned | the time to death from any cause |
| Scher ^11^ | 2016 | 50% or greater decline from baseline at 12 weeks | radiographic progression was determined by independent blinded review of available radionuclide bone scans, CTs, or MRIs, using the PCWG2 criteria, and calculated from therapy initiation until radiologically confirmed progression or death owing to any cause within 60 days of stopping treatment. | was calculated from initiation of therapy to death from any cause. |
| Seitz ^12^ | 2017 | a PSA level decline of 50% | worsening of disease related symptoms or new cancer-related complications, radiographic progression according to RECIST, two or more new bone lesions on bone scan, or death, whichever occurred first | the time to death from any cause |
| Sharp ^13^ | 2019 | 50% or greater decline from baseline at 12 weeks | time from start of therapy to documented radiological progression or clinical progression (including change of therapy, addition of investigational medicinal product, or stopping of treatment) | time from start of therapy to date of death or last follow-up/contact |
| Sieuwerts ^14^ | 2019 | 50% decline from baseline to 12 weeks | not mentioned | the time to death from any cause |
| Steinestel ^15^ | 2015 | ≥50% PSA decline at any time on therapy, maintained for ≥4 weeks | not mentioned | not mentioned |
| Tagawa ^16^ | 2018 | at least a 50% (PSA50) reduction from baseline either by C5D1(prior to switch) | the time between randomization and the first documentation of radiographic tumor progression (using RECIST 1.1), clinical progression (including skeletal-related events, increasing pain requiring escalation of narcotic analgesics, urinary obstruction, etc.), PSA progression, or death from any cause | not mentioned |
| Takeuchi ^17^ | 2016 | maximum reduction rate of PSA | not mentioned | not mentioned |
| To ^18^ | 2018 | PSA decrease 50%, confirmed 3 weeks later | not mentioned | not mentioned |
| Todenhöfer ^19^ | 2016 | PSA decrease 50%, confirmed 3 weeks later | not mentioned | the time to death from any cause |
| Welti ^20^ | 2016 | ≥50% decline in PSA from baseline at 12 weeks. | not mentioned | was measured from the date of mCRPC biopsy to the date of last contact |
| Zhu ^21^ | 2017 | not mentioned | symptomatic progression (worsening disease-related symptoms or new cancer-related complications), radiologic progression (on CT scan, ≥20% enlargement in sum diameter of soft-tissue target lesions RECIST; on bone scan, ≥2 new bone lesions), or death, whichever occurred first. | not mentioned |

*PSA* prostate-specific antigen, *PFS* progression free survival, *OS* overall survival, *CT* Computed Tomography, *RECIST* Response Evaluation Criteria in Solid Tumors criteria, *MRI* Magnetic Resonance Imaging, *PCWG2* Prostate Cancer Working Group 2, *mCRPC* metastatic castration-resistant prostate cancer

**Table S2. Target simples and AR-V7 detection method of the included studies**

| **Study** | **Year** | **Target simples** | **AR-V7 detection method** | **AR-V7 detection assay** |
| --- | --- | --- | --- | --- |
| Antonarakis ^1^ | 2017 | CTC in PB | RT-PCR | CTC analyses were conducted using a modified AdnaTest platform. Isolation and enrichment of CTCs was performed using the ProstateCancerSelect kit. Custom primers were designed to detect full-length androgen receptor (AR-FL) mRNA and AR-V7 mRNA |
| Antonarakis ^2^ | 2015 | CTC in PB | RT-PCR | CTC analyses were conducted using a modified AdnaTest platform. Isolation and enrichment of CTCs was performed using the ProstateCancerSelect kit, and mRNA expression analyses were performed using the ProstateCancerDetect kit with multiplexed reverse-transcription polymerase chain reaction primers to establish the presence or absence of CTCs. Custom primers were used to detect the full-length AR (ARFL) mRNA and AR-V7 mRNA |
| Antonarakis ^3^ | 2014 | CTC in PB | RT-PCR | CTC analyses were conducted using the commercially-available AlereTM CTC AdnaTest platform (AdnaGen, Langenhagen, Germany). Isolation and enrichment of CTCs was performed using the ProstateCancerSelect kit, and mRNA expression analyses were performed using the ProstateCancerDetect kit with multiplexed reverse-transcription polymerase-chain-reaction (qRT-PCR) primers to detect the presence of CTCs. Quantitative reverse-transcriptase–polymerase-chain-reaction (RT-PCR) assays were used for mRNA detection. |
| Armstrong ^4^ | 2019 | CTC in PB | RT-PCR | CTC analyses were conducted using a modified AdnaTest platform. Isolation and enrichment of CTCs was performed using the ProstateCancerSelect kit. Custom primers were designed to detect full-length androgen receptor (AR-FL) mRNA and AR-V7 mRNA. |
|  |  |  | IFS | CTC from mmCRPC patient samples, underwent automated immunofluorescent staining for DNA, cytokeratins (CK), CD45 (lymphocyte common antigen), and AR-V7. A rabbit monoclonal anti-AR-V7 antibody (EPR15656; Abcam) was used. |
| Del Re ^5^ | 2016 | Exosomes of plasma in PB | dd-PCR | Exosome isolation from plasma was performed using the exoRNeasy kit (Qiagen, Valencia, CA, USA) on exoEasy spin columns, and RNA was extracted from the vesicles bound to the silica membrane using the QIAzol phenol/guanidine-based lysis solution. The analysis of AR-V7 in RNA was performed by ddPCR using the One-Step RT-ddPCR kit |
| Nakazawa ^6^ | 2015 | CTC in PB | RT-PCR | Modified version of the AdnaTest platform for CTC analysis was used. CTCs were enriched from peripheral blood using the ProstateCancerSelect kit. mRNA expression analysis was conducted using the ProstateCancerDetect kit, along with multiplexed reverse-transcription polymerase chain reaction analyses using custom primers to detect full-length AR (AR-FL) mRNA and AR-V7 mRNA. |
| Okegawa ^7^ | 2018 | CTC in PB | RT-PCR | An on-chip multi-imaging flow cytometry system was developed to obtain morphometric parameters of cell clusters which could identify clustered CTCs in blood with the same level of precision using this system. CTC cells were made for each sample tested. The test was adapted for detection of AR-V7 by polymerase chain reaction (PCR)using custom primers specific for AR-V7 |
| Onstenk ^8^ | 2015 | CTC in PB | RT-PCR | CTC was processed using the CellSearch Profile Kit. After RNA isolation, cDNA generation, and preamplification, expression levels of AR-WT and AR-V7 were measured by RT-qPCR in an 11% aliquot of the original starting material using Taqman Gene Expression Assays |
| Qu ^9^ | 2016 | PBMC | dd-PCR | Total RNA from the peripheral blood mononuclear cell fraction was extracted with TRIzol reagent. Droplet generation, PCR reactions, and detection were carried out according to the manufacturer's instruction. |
| Scher ^10^ | 2018 | CTC in PB | IFS | CTC from mmCRPC patient samples, underwent automated immunofluorescent staining for DNA, cytokeratins (CK), CD45 (lymphocyte common antigen), and AR-V7. A rabbit monoclonal anti-AR-V7 antibody (EPR15656; Abcam) was used. |
| Scher ^11^ | 2016 | CTC in PB | IFS | CTC from mmCRPC patient samples, underwent automated immunofluorescent staining for DNA, cytokeratins (CK), CD45 (lymphocyte common antigen), and AR-V7. A rabbit monoclonal anti-AR-V7 antibody (EPR15656; Abcam) was used. |
| Seitz ^12^ | 2017 | PB | dd-PCR | RNA was extracted from blood samples according to the manufacturer’s instructions using the PAXgene blood RNA kit (Qiagen). AR-V7 mRNA levels were simultaneously quantified in a dual color assay using custom primer and hydrolysis probe sets on a QX200 ddPCR system with automatic droplet generation |
| Sharp ^13^ | 2019 | Biopsies | IHC | AR-V7 IHC was performed using recombinant rabbit monoclonal anti–AR-V7 antibody (Clone RM7, RevMAb Biosciences). AR-V7 protein expression was determined for each case by a pathologist blinded to clinical data using the modified H score (HS) method, a semiquantitative assessment of staining intensity that reflects antigen concentration. |
| Sieuwerts ^14^ | 2019 | CTC in PB | RT-PCR | CellSearch-enriched CTCs from the EDTA characterization tube were subjected to RNA isolation using the AllPrep DNA/RNA Micro Kit (Qiagen, Hilden, Germany) followed by cDNA generation and preamplification of individual genes. Individual transcript expression levels were measured using quantitative polymerase chain reaction (qPCR) |
| Steinestel ^15^ | 2015 | CTC in PB | RT-PCR | Isolation of RNA from CTCs employed the AdnaGen ProstateCancerDetect Kit (AdnaGen, Langenhagen, Germany). ΔCt method was applied to determine the relative AR-V7 expression levels. |
| Tagawa ^16^ | 2018 | CTC in PB | dd-PCR | Total RNA was extracted from the enriched CTCs pool using the RNAeasy Plus Micro kit (Qiagen) asper manufacturer’s instructions. After PCR, droplets that contained a template had a fluorescent signal (positive droplets) that distinguished them from the droplets without a template (negative droplets) |
| Takeuchi ^17^ | 2016 | PB | Nested PCR | Total RNA was extracted from 0.4 mL of whole blood using oligo dT primer and a DNA Extractor® WB-Rapid Kit (Wako Pure Chemical Industries, Ltd, Osaka, Japan). Complementary DNA was synthesized from total RNA using a PrimeScript™ II first strand cDNA Synthesis Kit (Takara Bio, Inc., Kusatsu, Japan). Thirty-five cycles of polymerase chain reactions (PCRs) (primary PCR) in 50 µL were performed using a Premix Taq DNA Polymerase (Takara Bio, Inc.) for the amplification of AR-V7. In addition, 30 cycles of nested PCRs for AR-V7, prototype AR, and PSA were conducted using 1 µL of primary PCR products by substituting each corresponding antisense primer. |
| To ^18^ | 2018 | PB | RT-PCR | RNA is isolated and subject to reverse transcription using a primer specific for the AR-V7 transcript. Quantitative polymerase chain reaction (qPCR) is then performed using Taqman chemistry, and a probe/primer is set for specifically targeted unique sequences within the AR-V7 transcript |
| Todenhöfer ^19^ | 2016 | CTC in PB | RT-PCR | Reverse transcription was performed using the Superscript Vilo cDNA synthesis kit (ThermoFisher Scientific, Waltham, MA, USA) according to the manufacturer’s protocol. Following gene specific preamplification, TaqMan gene expression assays (ThermoFisher Scientific, Waltham, MA, USA) were performed according to the manufacturer’s protocol. |
| Welti ^20^ | 2016 | Biopsies | IHC | IHC was performed using the rabbit AR-V7 monoclonal antibody clone EP343 (Epitomics). Cases were scored by a pathologist blinded to clinical data using the modified H score (HS) method, a semiquantitative assessment of staining intensity that reflects antigen concentration. |
| Zhu ^21^ | 2017 | Biopsies | RISH | The BaseScope assays (Advanced Cell Diagnostics, Inc., Hayward, CA) for AR-V7 were developed to achieve junction-specific detection of the AR transcripts. The BaseScope assay is based on the RNAscope technology but uses an additional signal amplification step and requires only one “double Z” (1 ZZ) probe pair for single-molecule detection. |

*AR-V7* androgen receptor splice variant-7, *CTC* circulating tumor cell, *PB* peripheral blood, *RT-PCR* reverse-transcription polymerase chain reaction, *IFS* immunofluorescent staining, *dd-PCR* droplet-digital polymerase chain reaction, *cDNA* complementary deoxyribonucleic acid, *PBMC* peripheral blood mononuclear cell, *mCRPC* metastatic castration-resistant prostate cancer, *IHC* immunohistochemistry, *PCR* polymerase chain reaction, *RISH* RNA in situ hybridization, *PSA* prostate-specific antigen.

**Table S3. PSA response rate of mCRPC patients treated with ARSi in different AR-V7 status**

| **Author** | **Year** | **method** | **treatment** | **AR-V7 negative with PSA response** | **AR-V7 negative without PSA response** | **AR-V7 positive with PSA response** | **AR-V7 positive without PSA response** |
| --- | --- | --- | --- | --- | --- | --- | --- |
| Antonarakis ^1^ | 2017 | RT-PCR | abiraterone or enzalutamide | 59 | 54 | 5 | 31 |
| Antonarakis ^3^ | 2014 | RT-PCR | abiraterone or enzalutamide | 27 | 17 | 0 | 18 |
| Armstrong (ES) ^4^ | 2019 | IFS | abiraterone or enzalutamide | 26 | 68 | 0 | 11 |
| Armstrong (JHU) ^4^ | 2019 | RT-PCR | abiraterone or enzalutamide | 28 | 60 | 11 | 17 |
| Del Re ^5^ | 2016 | dd-PCR | abiraterone or enzalutamide | 14 | 8 | 1 | 13 |
| Nakazawa ^6^ | 2015 | RT-PCR | abiraterone or enzalutamide | 2 | 2 | 0 | 3 |
| Okegawa ^7^ | 2018 | RT-PCR | abiraterone or enzalutamide | 10 | 13 | 4 | 22 |
| Scher ^11^ | 2016 | IFS | abiraterone or enzalutamide | 47 | 65 | 0 | 16 |
| Seitz ^12^ | 2017 | dd-PCR | abiraterone or enzalutamide | 31 | 31 | 0 | 12 |
| Sharp^13^ | 2019 | IHC | abiraterone or enzalutamide | 8 | 0 | 15 | 13 |
| Steinestel ^15^ | 2015 | RT-PCR | abiraterone or enzalutamide | 5 | 3 | 1 | 13 |
| Takeuchi ^17^ | 2016 | Nested PCR | abiraterone or enzalutamide | 1 | 4 | 0 | 6 |
| To ^18^ | 2018 | RT-PCR | abiraterone or enzalutamide | 20 | 10 | 4 | 3 |
| Todenhöfer ^19^ | 2016 | RT-PCR | abiraterone | 13 | 18 | 0 | 4 |
| Welti ^20^ | 2016 | IHC | abiraterone | 5 | 6 | 0 | 4 |
| Zhu ^21^ | 2017 | RISH | abiraterone or enzalutamide | 9 | 4 | 5 | 7 |
| **Total** | - | - | - | 305 | 363 | 46 | 193 |

*PSA* prostate-specific antigen, *mCRPC* metastatic castration-resistant prostate cancer, *ARSi* androgen receptor signaling inhibitors, *AR-V7* androgen receptor splice variant-7, *RT-PCR* reverse-transcription polymerase chain reaction, *IFS* immunofluorescent staining, *dd-PCR* droplet-digital polymerase chain reaction, *IHC* immunohistochemistry, *PCR* polymerase chain reaction, *RISH* RNA in situ hybridization.

**Table S4. PSA response rate of mCRPC patients treated with taxane in different AR-V7 status**

| **Author** | **Year** | **method** | **treatment** | **AR-V7 negative with PSA response** | **AR-V7 negative without PSA response** | **AR-V7 positive with PSA response** | **AR-V7 positive without PSA response** |
| --- | --- | --- | --- | --- | --- | --- | --- |
| Sharp (Cohort 1) ^13^ | 2019 | IHC | docetaxel | 13 | 7 | 7 | 10 |
| Sharp (Cohort 2) ^13^ | 2019 | IHC | docetaxel | 1 | 1 | 3 | 0 |
| Sieuwerts ^14^ | 2019 | RT-PCR | cabazitaxel | 2 | 12 | 1 | 14 |
| Antonarakis ^2^ | 2015 | RT-PCR | docetaxel/cabazitaxel | 17 | 28 | 6 | 12 |
| Nakazawa ^6^ | 2015 | RT-PCR | taxane | 7 | 11 | 10 | 27 |
| Onstenk ^8^ | 2015 | RT-PCR | cabazitaxel | 10 | 7 | 8 | 29 |
| Tagawa ^16^ | 2018 | dd-PCR | docetaxel/cabazitaxel | 10 | 17 | 5 | 20 |
| Scher ^11^ | 2016 | IFS | taxane | 11 | 7 | 13 | 23 |
| **Total** | - | - | - | 71 | 90 | 53 | 135 |

*PSA* prostate-specific antigen, *mCRPC* metastatic castration-resistant prostate cancer, *ARSi* androgen receptor signaling inhibitors, *AR-V7* androgen receptor splice variant-7, *IHC* immunohistochemistry, *RT-PCR* reverse-transcription polymerase chain reaction, *dd-PCR* droplet-digital polymerase chain reaction, *IFS* immunofluorescent staining, *RISH* RNA in situ hybridization.

**Table S5 Quality assessment of included studies**

| **Study** | **Year** | **Study design** | **Selection** | **Comparability** | **Outcome** | **Total score** |
| --- | --- | --- | --- | --- | --- | --- |
| Antonarakis ^1^ | 2017 | Prospective cohort study | **** | * | *** | 8 |
| Antonarakis ^2^ | 2015 | Prospective cohort study | **** | ** | *** | 9 |
| Antonarakis ^3^ | 2014 | Prospective cohort study | **** | ** | *** | 9 |
| Armstrong ^4^ | 2019 | Prospective cohort study | *** | ** | *** | 8 |
| Del Re ^5^ | 2016 | Prospective cohort study | *** | ** | *** | 8 |
| Nakazawa ^6^ | 2015 | Prospective cohort study | *** | ** | ** | 7 |
| Okegawa ^7^ | 2018 | Retrospective cohort study | *** | * | *** | 7 |
| Onstenk ^8^ | 2015 | Prospective cohort study | *** | ** | ** | 7 |
| Qu ^9^ | 2016 | Retrospective cohort study | *** | ** | ** | 7 |
| Scher ^10^ | 2018 | Prospective cohort study | **** | ** | ** | 8 |
| Scher ^11^ | 2016 | Prospective cohort study | *** | ** | ** | 7 |
| Seitz ^12^ | 2017 | Prospective cohort study | *** | * | *** | 7 |
| Sharp ^13^ | 2019 | Retrospective cohort study | **** | ** | ** | 8 |
| Sieuwerts ^14^ | 2019 | Prospective cohort study | *** | ** | ** | 7 |
| Steinestel ^15^ | 2015 | Prospective cohort study | *** | ** | ** | 7 |
| Tagawa ^16^ | 2018 | Prospective cohort study | *** | * | ** | 6 |
| Takeuchi ^17^ | 2016 | Retrospective cohort study | ** | * | ** | 5 |
| To ^18^ | 2018 | Prospective cohort study | ** | ** | ** | 6 |
| Todenhöfer ^19^ | 2016 | Prospective cohort study | *** | * | ** | 6 |
| Welti ^20^ | 2016 | Retrospective cohort study | *** | * | ** | 6 |
| Zhu ^21^ | 2017 | Retrospective cohort study | *** | ** | *** | 8 |

**Part 4 Supplementary Figures**

**Figure S1**. **Sensitivity analysis of AR-V7 status in predicting PSA response of the mCRPC patients treated with ARSi**.


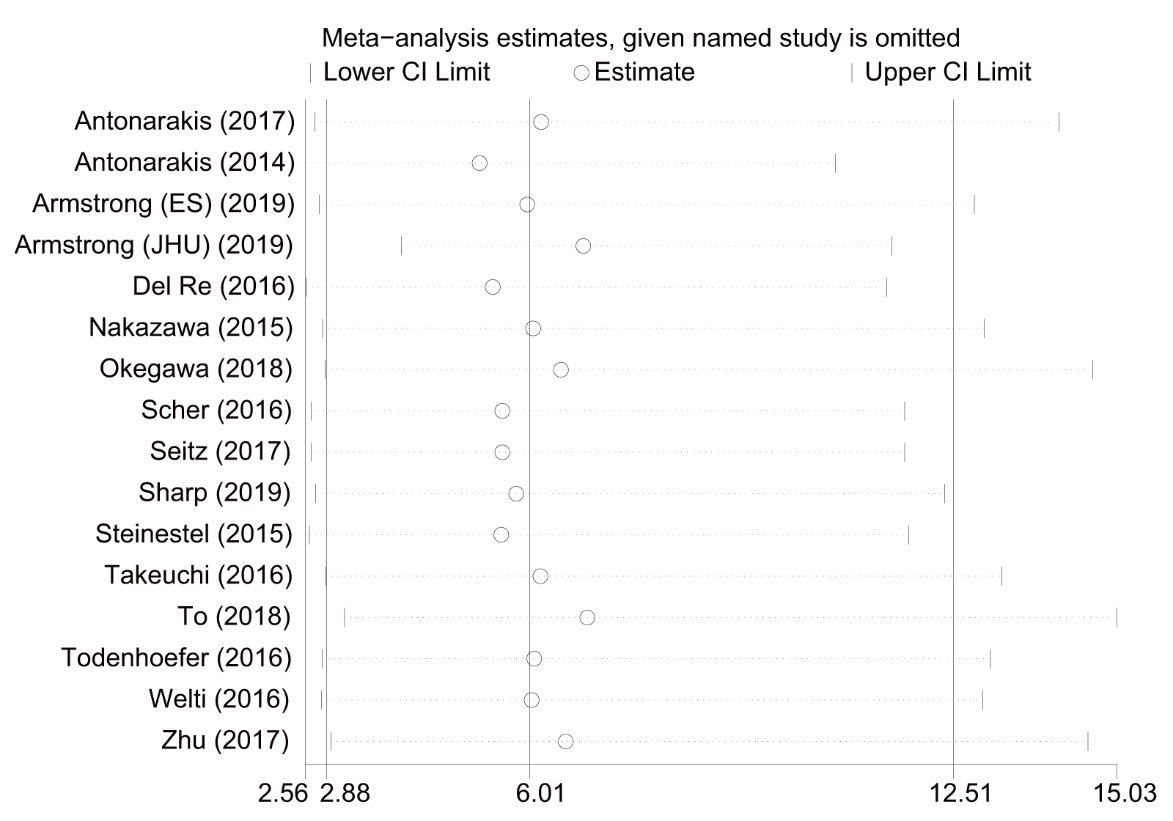


Compared with AR-V7 negative patients, AR-V7-positive patients had lower PSA response (OR 6.01, 95% CI 2.88-12.51). Sensitivity analysis was applied to evaluate the stability of the results. During the analysis each study was removed at a time and the rest were used to recalculate the pooled OR to find whether the results were affected statistically significant. OR with 95% CI had a little change after the removal of any studies, confirming the stability of the results.

**Supplementary Figure S2**. **Publication bias of studies the reported PSA response of mCRPC patients treated with ARSi.** (A) Begg’s funnel plot; (B) Egger’s linear regression plot.


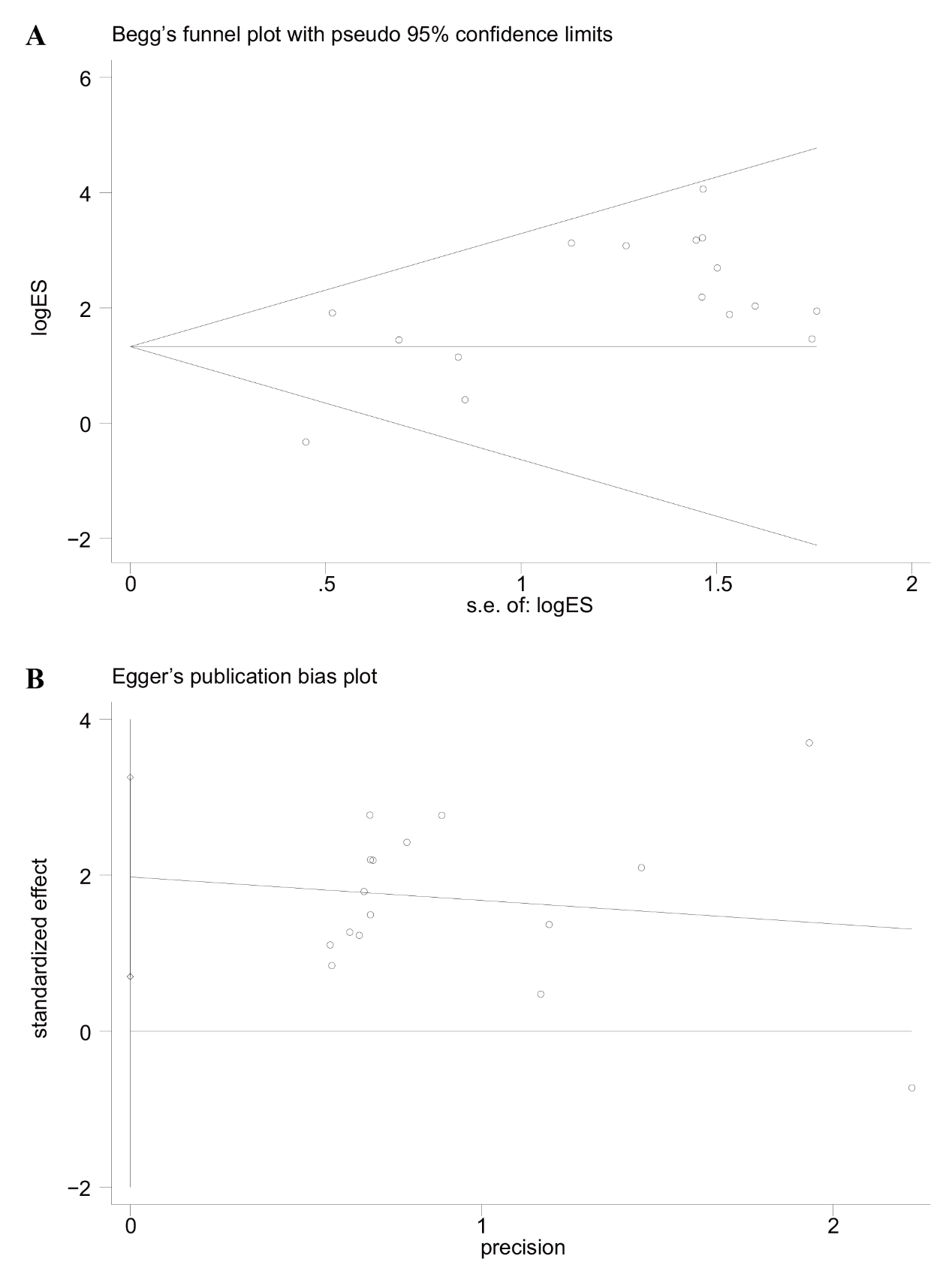


**Figure S3. Publication bias of studies the reported PFS of mCRPC patients treated with ARSi.** (A) Begg’s funnel plot; (B) Egger’s linear regression plot.


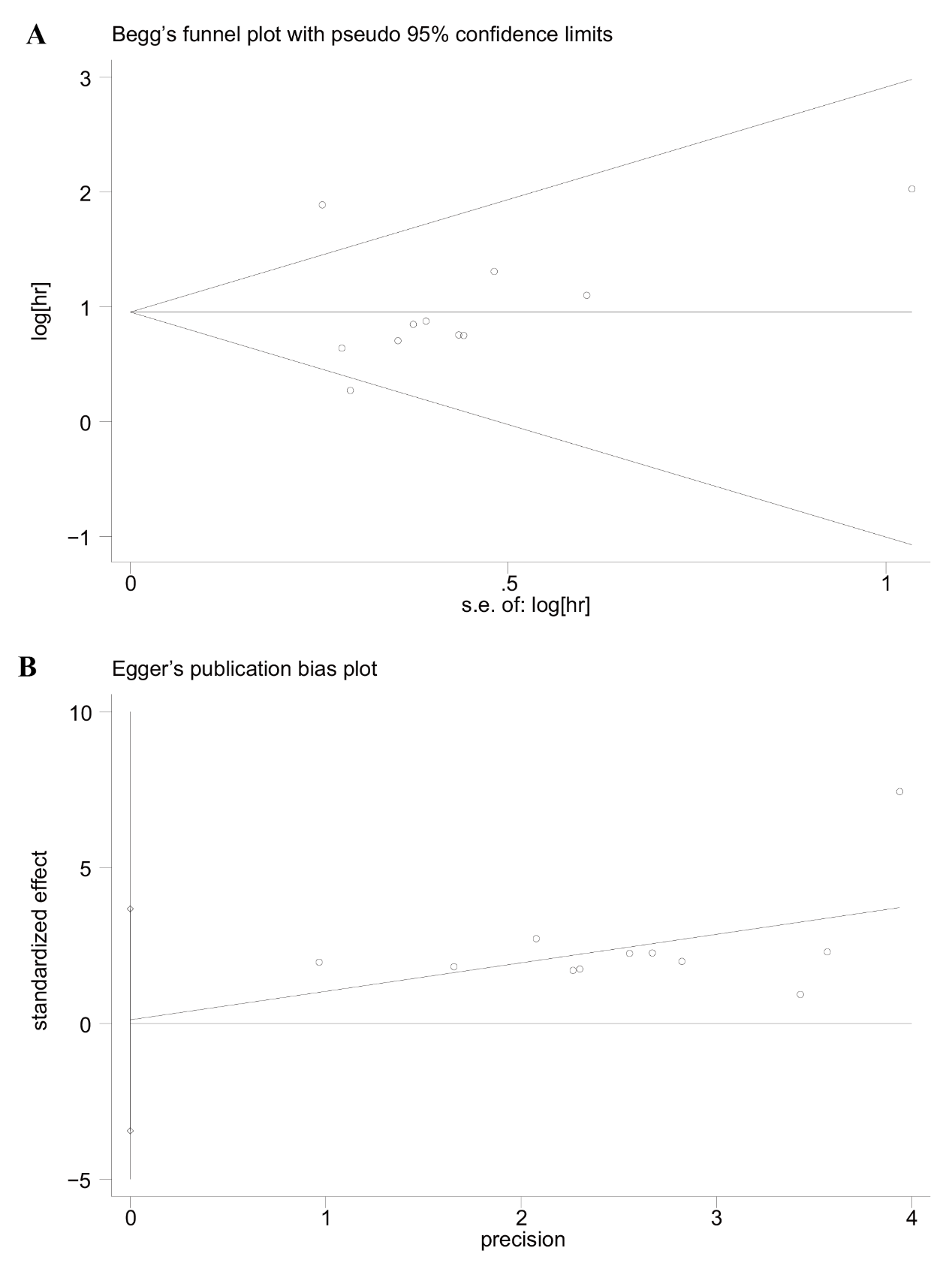


**Figure S4. Publication bias of studies the reported OS of mCRPC patients treated with ARSi.** (A) Begg’s funnel plot; (B) Egger’s linear regression plot.


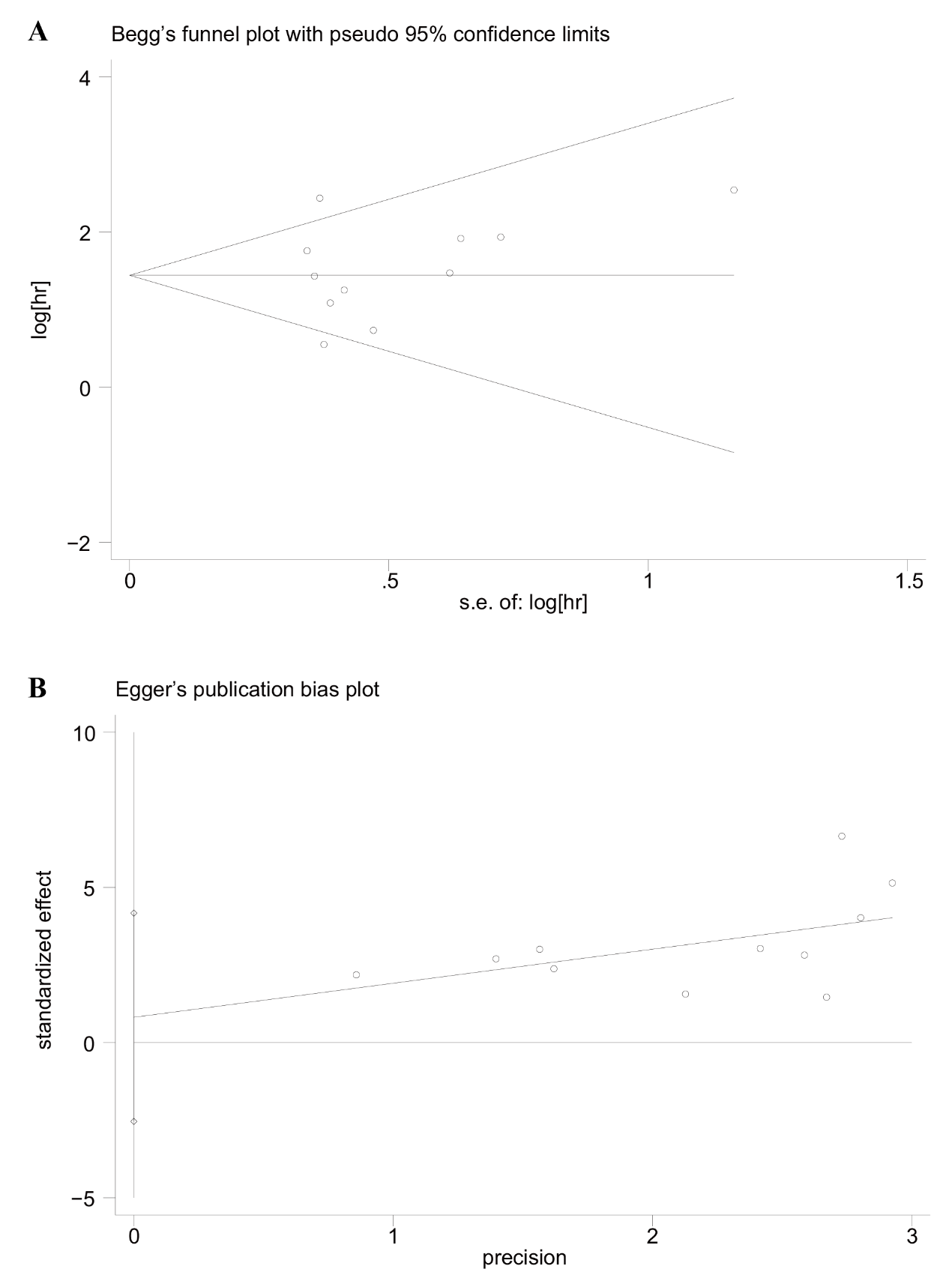


**Part 5 References**

1. Antonarakis ES, Lu C, Luber B, et al. Clinical Significance of Androgen Receptor Splice Variant-7 mRNA Detection in Circulating Tumor Cells of Men With Metastatic Castration-Resistant Prostate Cancer Treated With First- and Second-Line Abiraterone and Enzalutamide. *Journal of clinical oncology : official journal of the American Society of Clinical Oncology* 2017; **35**(19): 2149-56.

2. Antonarakis ES, Lu C, Luber B, et al. Androgen Receptor Splice Variant 7 and Efficacy of Taxane Chemotherapy in Patients With Metastatic Castration-Resistant Prostate Cancer. *JAMA Oncol* 2015; **1**(5): 582-91.

3. Antonarakis ES, Lu C, Wang H, et al. AR-V7 and resistance to enzalutamide and abiraterone in prostate cancer. *N Engl J Med* 2014; **371**(11): 1028-38.

4. Armstrong AJ, Halabi S, Luo J, et al. Prospective Multicenter Validation of Androgen Receptor Splice Variant 7 and Hormone Therapy Resistance in High-Risk Castration-Resistant Prostate Cancer: The PROPHECY Study. *Journal of clinical oncology : official journal of the American Society of Clinical Oncology* 2019; **37**(13): 1120-9.

5. Del Re M, Biasco E, Crucitta S, et al. The Detection of Androgen Receptor Splice Variant 7 in Plasma-derived Exosomal RNA Strongly Predicts Resistance to Hormonal Therapy in Metastatic Prostate Cancer Patients. *Eur Urol* 2017; **71**(4): 680-7.

6. Nakazawa M, Lu C, Chen Y, et al. Serial blood-based analysis of AR-V7 in men with advanced prostate cancer. *Ann Oncol* 2015; **26**(9): 1859-65.

7. Okegawa T, Ninomiya N, Masuda K, Nakamura Y, Tambo M, Nutahara K. AR-V7 in circulating tumor cells cluster as a predictive biomarker of abiraterone acetate and enzalutamide treatment in castration-resistant prostate cancer patients. *Prostate* 2018; **78**(8): 576-82.

8. Onstenk W, Sieuwerts AM, Kraan J, et al. Efficacy of Cabazitaxel in Castration-resistant Prostate Cancer Is Independent of the Presence of AR-V7 in Circulating Tumor Cells. *Eur Urol* 2015; **68**(6): 939-45.

9. Qu F, Xie W, Nakabayashi M, et al. Association of AR-V7 and Prostate-Specific Antigen RNA Levels in Blood with Efficacy of Abiraterone Acetate and Enzalutamide Treatment in Men with Prostate Cancer. *Clin Cancer Res* 2017; **23**(3): 726-34.

10. Scher HI, Graf RP, Schreiber NA, et al. Assessment of the Validity of Nuclear-Localized Androgen Receptor Splice Variant 7 in Circulating Tumor Cells as a Predictive Biomarker for Castration-Resistant Prostate Cancer. *JAMA Oncol* 2018; **4**(9): 1179-86.

11. Scher HI, Lu D, Schreiber NA, et al. Association of AR-V7 on Circulating Tumor Cells as a Treatment-Specific Biomarker With Outcomes and Survival in Castration-Resistant Prostate Cancer. *JAMA Oncol* 2016; **2**(11): 1441-9.

12. Seitz AK, Thoene S, Bietenbeck A, et al. AR-V7 in Peripheral Whole Blood of Patients with Castration-resistant Prostate Cancer: Association with Treatment-specific Outcome Under Abiraterone and Enzalutamide. *Eur Urol* 2017; **72**(5): 828-34.

13. Sharp A, Coleman I, Yuan W, et al. Androgen receptor splice variant-7 expression emerges with castration resistance in prostate cancer. *J Clin Invest* 2019; **129**(1): 192-208.

14. Sieuwerts AM, Onstenk W, Kraan J, et al. AR splice variants in circulating tumor cells of patients with castration-resistant prostate cancer: relation with outcome to cabazitaxel. *Mol Oncol* 2019; **13**(8): 1795-807.

15. Steinestel J, Luedeke M, Arndt A, et al. Detecting predictive androgen receptor modifications in circulating prostate cancer cells. *Oncotarget* 2019; **10**(41): 4213-23.

16. Tagawa ST, Antonarakis ES, Gjyrezi A, et al. Expression of AR-V7 and ARv(567es) in Circulating Tumor Cells Correlates with Outcomes to Taxane Therapy in Men with Metastatic Prostate Cancer Treated in TAXYNERGY. *Clin Cancer Res* 2019; **25**(6): 1880-8.

17. Takeuchi T, Okuno Y, Hattori-Kato M, Zaitsu M, Mikami K. Detection of AR-V7 mRNA in whole blood may not predict the effectiveness of novel endocrine drugs for castration-resistant prostate cancer. *Res Rep Urol* 2016; **8**: 21-5.

18. To SQ, Kwan EM, Fettke HC, et al. Expression of Androgen Receptor Splice Variant 7 or 9 in Whole Blood Does Not Predict Response to Androgen-Axis-targeting Agents in Metastatic Castration-resistant Prostate Cancer. *Eur Urol* 2018; **73**(6): 818-21.

19. Todenhofer T, Azad A, Stewart C, et al. AR-V7 Transcripts in Whole Blood RNA of Patients with Metastatic Castration Resistant Prostate Cancer Correlate with Response to Abiraterone Acetate. *J Urol* 2017; **197**(1): 135-42.

20. Welti J, Rodrigues DN, Sharp A, et al. Analytical Validation and Clinical Qualification of a New Immunohistochemical Assay for Androgen Receptor Splice Variant-7 Protein Expression in Metastatic Castration-resistant Prostate Cancer. *Eur Urol* 2016; **70**(4): 599-608.

21. Zhu Y, Sharp A, Anderson CM, et al. Novel Junction-specific and Quantifiable In Situ Detection of AR-V7 and its Clinical Correlates in Metastatic Castration-resistant Prostate Cancer. *Eur Urol* 2018; **73**(5): 727-35.
